# Erythema Ab Igne Associated With Prolonged Direct Skin Contact With a Space Heater: A Case Report

**DOI:** 10.7759/cureus.83204

**Published:** 2025-04-29

**Authors:** Viniya Patidar, Mary Ann Kirkconnell Hall, Bonnie Cruser

**Affiliations:** 1 Division of Hospital Medicine, Emory University School of Medicine, Atlanta, USA

**Keywords:** burns, erythema, erythema ab igne, heating, reticulated rash, wounds and injuries

## Abstract

Erythema ab igne is a rare condition manifesting in a reticulated, hyperpigmented rash that develops from chronic exposure to direct heat or infrared radiation. Diagnosis is clinical, with biopsy only required when malignancy is suspected. Historically, individuals repeatedly exposed to direct heat, such as bakers and metalworkers, were at increased risk, but since the development of central heating, incidence has significantly decreased in developed countries. However, it is important to recognize that erythema ab igne may occur with prolonged chronic exposure to normally functioning heated items such as car seats and, in our case, a space heater. The mainstay of treatment is the removal of heat exposure; delays in diagnosis and ongoing heat exposure can result in permanent pigmentation or progression to malignant transformation, leading to Merkel cell carcinoma, basal cell carcinoma, and squamous cell carcinoma.

We describe a case of a woman who presented with complaints of cramping pain in her legs with discoloration and a lacy rash, particularly around her calves and moving upward toward her inner legs, who we determined had erythema ab igne caused by multi-hour direct exposure of her skin to a space heater placed in between her legs on a daily basis for the past few months. The treatment recommended was to avoid ongoing heat exposure; the patient was counseled extensively on the importance of stopping the use of space heaters for prolonged periods of time near her legs and discharged in stable condition.

## Introduction

In the original Latin, erythema ab igne literally means "redness from fire." Erythema ab igne is a rarely reported condition where a reticulated, hyperpigmented rash is caused by chronic exposure to either direct heat or infrared radiation [1]. It closely resembles livedo reticularis, which is associated with underlying systemic illness and requires additional workup and treatment. It can also mimic other dermatologic conditions, further complicating the diagnosis.

"Retiform" and "reticular" are synonyms that mean "net-like." The differential diagnosis for a reticular rash includes other medical conditions such as livedo reticularis, cutis marmorata, livedo racemosa, poikiloderma of Civatte, or COVID-associated rash [2]. Erythema ab igne is usually asymptomatic; however, some patients report itching or burning involving the rash site [3]. It can initially start off as an erythematous patch and eventually can progress to involve skin pigment changes and fibrosis in advanced, chronic cases. The diagnosis is largely clinical, not requiring a skin biopsy other than in cases where malignancy is suspected [1-3].

In premodern times, erythema ab igne was most frequently observed in individuals repeatedly exposed to direct heat, often occupationally, such as bakers and metalworkers, or by proximity to sources of heating such as open fires. Its incidence has significantly decreased in developed countries since the introduction of central heating and subsequent reduction in skin exposure to direct heat sources. There are still rare cases reported in individuals exposed to space heaters, laptop computers on bare skin, heated car seats, heating pads, and heated water bottles [2,4-6]. Most seat heaters have an upper limit of 43°C; however, malfunctioning seats can sometimes reach even higher levels, nearing around 48.9°C, and can cause second-degree burns, particularly in patients with impaired mobility and sensation. It is also important to recognize that erythema ab igne may occur with prolonged chronic exposure to normally functioning heated elements [5].

The primary approach to treatment is removing exposure to heat; delays in diagnosis and continued heat exposure may result in permanent pigmentation or progression to malignant transformation, leading to Merkel cell carcinoma, basal cell carcinoma, and squamous cell carcinoma [1,3].

In this report, we describe the case of a woman who presented with complaints of cramping pain in her legs, accompanied by discoloration and a lacy rash, particularly on the inner aspects of both upper thighs and down her calves. Subsequent investigation revealed that she had exposed her skin to a space heater placed between her legs for multiple hours each day for the previous several months, and she was diagnosed with erythema ab igne.

## Case presentation

Patient information

A 37-year-old woman with no known past medical history presented with several weeks' history of worsening crampy pain in her legs; she reported taking nonsteroidal anti-inflammatory drugs for pain with little relief. She had also noticed discoloration and a lacy rash, particularly around her calves and moving upward toward her inner legs. She reported feeling cold in her legs, further contributing to her discomfort, along with increased fatigue, lightheadedness, and dyspnea on exertion during this time.

Clinical findings

On presentation, her vitals were a temperature of 36.4°C, heart rate of 101, blood pressure of 125/77 mmHg, and oxygen saturation of 100% on room air. She denied any signs of bleeding and reported normal menstrual cycles. Results of an extended anemia panel suggested iron deficiency anemia (Table 1). In the context of her symptoms, anemia raised our clinical suspicion of an underlying autoimmune condition, and we ordered haptoglobin, lactate dehydrogenase, estimated sedimentation rate (ESR), C-reactive protein (CRP), HIV, and hepatitis studies (Table 1). All were within (or very near) normal values, with the exception of ESR and CRP, which were mildly elevated (Table 1).

**Table 1:** Laboratory values on presentation

Assay	Value	Reference range
Hemoglobin	6.8 g/dL	12.1-15.1 g/dL
Iron	32 mcg/dL	60-160 mcg/dL
Ferritin	3 ng/mL	10-150 ng/mL
Total iron-binding capacity	437 mcg/dL	240-450 mcg/dL
Transferrin saturation	7%	15%-50%
Haptoglobin	204 mg/dL	83-267 mg/dL
Lactate dehydrogenase	228 units/L	80-225 units/L
Estimated sedimentation rate	85 mm/hour	0-20 mm/hour
C-reactive protein	6.2 mg/dL	Normal: <0.3 mg/dL, normal or minor elevation: 0.3-1.0 mg/dL, moderate elevation: 1.0-10.0 mg/dL, marked elevation: >10.0 mg/dL, severe elevation: >50.0 mg/dL [7]
HIV	Negative	N/A
Acute viral hepatitis A, B, C	Negative	N/A

**Figure 1:**
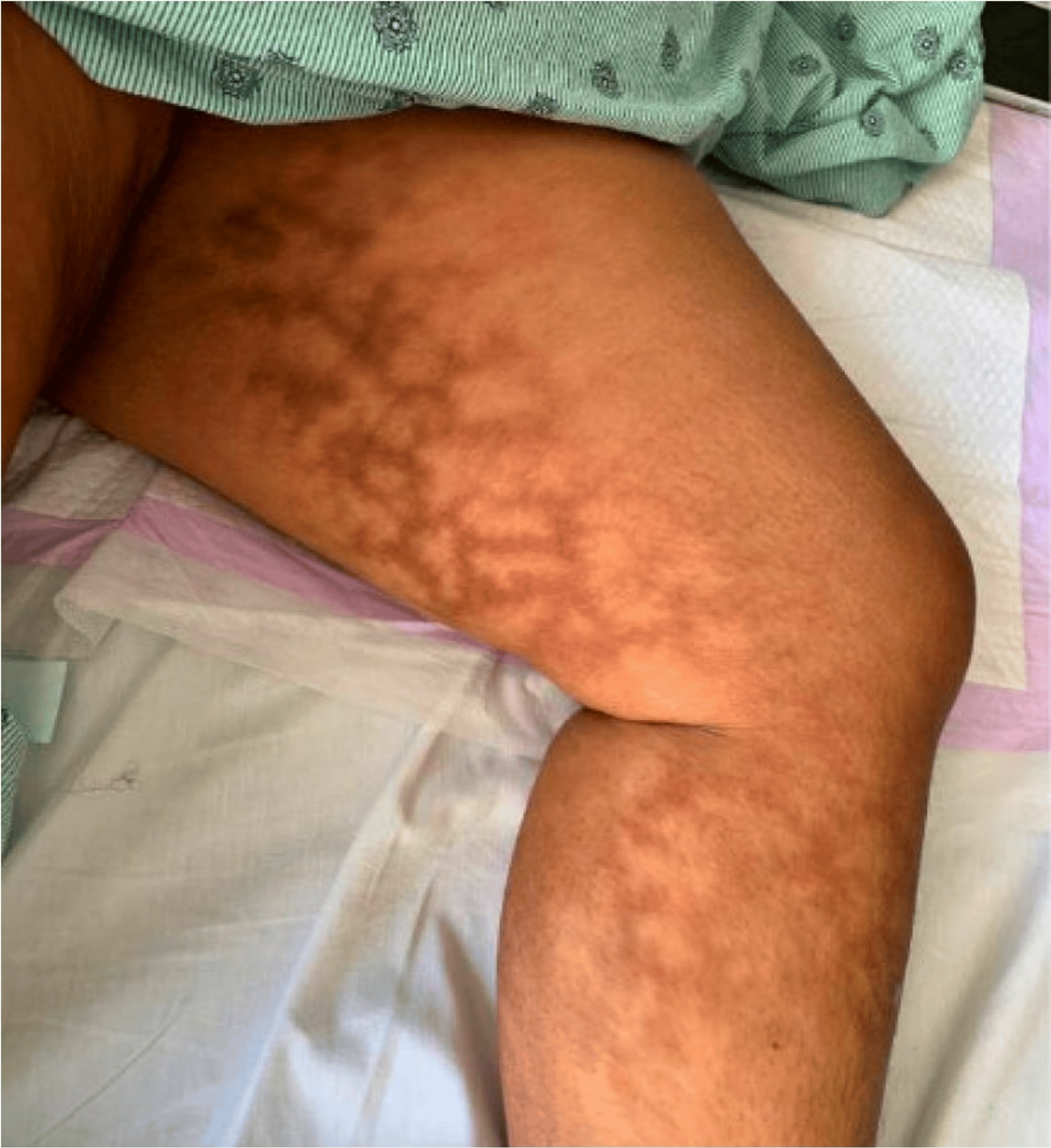
Erythema ab igne on the left inner thigh and calf

**Figure 2:**
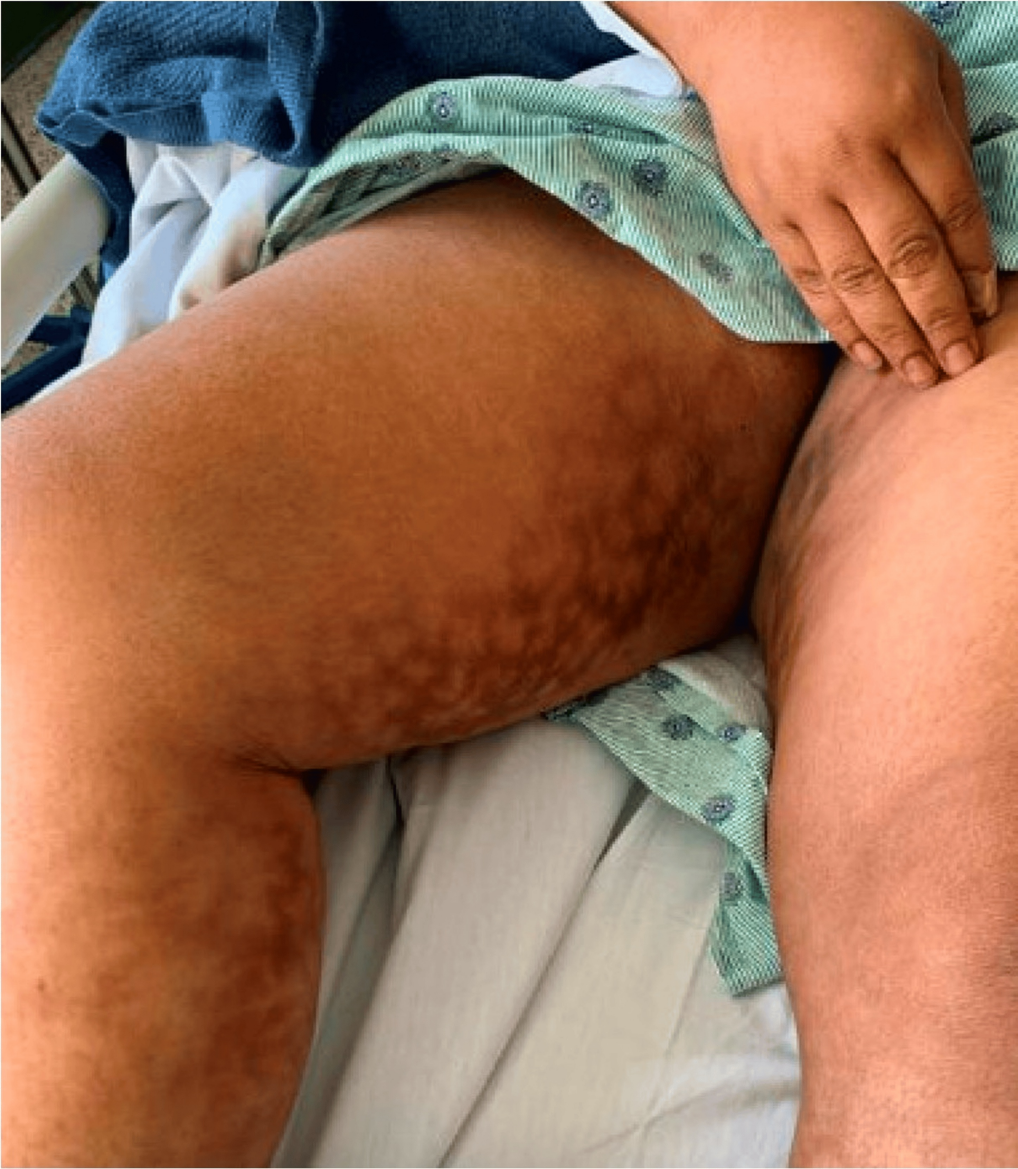
Erythema ab igne on the right inner thigh and calf

On examination, she had extensive mottling of skin with hyperpigmented changes involving her bilateral lower extremities, notably her calves and the inner aspects and back of her thighs (Figure 1 and Figure 2).

Diagnostic assessment

Due to her low hemoglobin level, the patient was admitted and transfused one unit of packed red blood cells and hemoglobin with an appropriate response.

For the lower extremity rash, we were concerned about livedo reticularis and consulted dermatology. Dermatology determined that her severe iron deficiency anemia was causing restless leg syndrome and pain. Upon further history obtained by dermatology, the patient revealed that due to her legs feeling cold and cramping, she had been placing a space heater between her legs with direct exposure of her skin for several hours a day, every day, for several months.

Due to this history of routine, prolonged, direct heat to the inner thighs and a clinical finding of fixed, brown retiform patches and thin plaques isolated to the area, the diagnosis was thought to be consistent with erythema ab igne, and it was determined that no additional autoimmune workup was needed.

Therapeutic intervention

The treatment recommended by dermatology was to avoid ongoing heat exposure. The patient was counseled extensively on the importance of stopping the use of space heaters for prolonged periods of time near her legs.

Follow-up and outcomes

The patient was discharged in stable condition but did not return to the clinic thereafter; hence, resolution of the rash was not visualized.

## Discussion

This case highlights the importance of clinical awareness and consideration of erythema ab igne. The differential diagnosis for a reticulated rash is limited. A history of heat exposure is sufficient to make a clinical diagnosis, thus avoiding the time and expense of further workup of more sinister causes, such as livedo reticularis, ruled out in our case by dermatology's definitive diagnosis, that can be associated with autoimmune illnesses.

Early detection and removal of the offending heat source(s) is important to prevent progression to permanent hyperpigmentation or carcinoma [6]. Erythema ab igne is twice as frequently diagnosed in women as in men and is more likely to occur involving the lower extremities; it can occur at any age but is most common between the ages of 40 and 70 years [7]. It is generally asymptomatic but can be associated with burning or itching [8].

Erythema ab igne is caused by thermal damage from heat sources not hot enough to cause burns, such as heated car seats, space heaters, or laptops [3,4]. Although the pathophysiology is not definitively known, it is thought that damage to superficial blood vessels results in hemosiderin deposition [7]. Erythema ab igne is not typically associated with the formation of bullae; however, there has been a reported case of bullous erythema ab igne, and it is thus important to include it in the differential diagnosis of bullous disorders [2].

As noted in the Introduction, the differential diagnosis for reticular rash includes erythema ab igne as well as livedo reticularis, livedo racemosa, cutis marmorata, poikiloderma of Civatte, COVID-associated rash, or drug-induced reticulate hyperpigmentation (Table 2). Livedo reticularis and cutis marmorata are typically worsened by cold, in contrast to erythema ab igne, which is worsened by heat [2]. Unlike livedo reticularis and livedo racemosa, erythema ab igne does not require laboratory evaluation for an underlying vasculitis or hypercoagulable state [2].

**Table 2:** Differential diagnosis of reticular rash

	Requires workup for autoimmune/hypercoagulable state?	Temperature association	Other notes
Erythema ab igne	No	Heat	-
Livedo reticularis	Yes, associated with lupus, cryoglobulinemia, and Raynaud phenomenon	Cold	-
Livedo racemosa	Yes	-	-
Cutis marmorata	No	Cold	Common in infants
Poikiloderma of Civatte	No	Heat	Associated with UV exposure
COVID-associated rash	No	-	-
Drug-induced reticulate hyperpigmentation	No	-	-

Erythema ab igne is a clinical diagnosis. Biopsy is typically not required to make a diagnosis of erythema ab igne [9,10] but should be done if there are papular or nodular lesions within the rash concerning for malignant transformation [1,10]. In our case, although our patient had mildly elevated ESR and CRP, dermatology was confident in the clinical diagnosis of erythema ab igne. We believe that the diagnosis was correct, supported by the fact that the patient did not present again for treatment in our health system.

Early diagnosis and removal of the offending heat source(s) is important, as, rarely, erythema ab igne has been reported to progress to squamous cell carcinoma, cutaneous marginal zone lymphoma, poorly differentiated carcinoma, and Merkel cell carcinoma [1,3,9,10]. The mainstay of treatment of erythema ab igne is removal of the heat source, which may reverse the skin changes of hyperpigmentation [1,3,9,11]. Consideration of the underlying causes of chronic pain, thus prompting the use of heat sources, is prudent. In some cases, if removal of the heat source does not fully or only minimally improve the rash, topical steroids, hydroquinone, 5-fluorouracil, or tretinoin may aid in reducing the discoloration [12].

## Conclusions

A reticular rash is easily recognizable on examination and has a limited differential diagnosis. In cases of erythema ab igne, a good history of heat exposure can yield the correct diagnosis without the need for biopsy. The most important diagnosis to rule out is livedo reticularis, which can be a sign of a systemic medium-vessel vasculitis. Removal of the offending heat source is important, as in rare cases, erythema ab igne can evolve to permanent skin discoloration or carcinoma.
